# Serum uric acid to creatinine ratio is a useful predictor of all-cause mortality among hypertensive patients

**DOI:** 10.1186/s40885-023-00235-8

**Published:** 2023-04-01

**Authors:** Ryuichi Kawamoto, Asuka Kikuchi, Daisuke Ninomiya, Yoshio Tokumoto, Teru Kumagi

**Affiliations:** 1 Department of Community Medicine, Ehime University Graduate School of Medicine, Toon, Japan; 2 Department of Internal Medicine, Seiyo Municipal Nomura Hospital, Seiyo, Japan

**Keywords:** Uric acid, Creatinine, Mortality rate, Risk factors, Hypertension

## Abstract

**Background:**

Many of the existing research studies have shown that serum uric acid (SUA) is a predictor of renal disease progression. More recently, studies have suggested an association between renal function-normalized SUA and all-cause mortality in adults. This study aims to examine the association between the ratio of SUA to creatinine (SUA/Cr) and all-cause mortality with a focus on hypertensive patients.

**Methods:**

This study is based on 2,017 participants, of whom 916 were male (mean age, 67 ± 11 years) and 1,101 were female (mean age, 69 ± 9 years). All participants were part of the Nomura Cohort Study in 2002 (cohort 1) and 2014 (cohort 2), as well as the follow-up period (2002 follow-up rate, 94.8%; 2014 follow-up rate, 98.0%). We obtained adjusted relative risk estimates for all-cause mortality from a basic resident register. In addition, we employed a Cox proportional hazards model and adjusted it for possible confounders to determine the hazard ratio (HR) and 95% confidence interval (CI).

**Results:**

Of the total participants, 639 (31.7%) were deceased; of these, 327 (35.7%) were male and 312 (28.3%) were female. We found an independent association between a higher ratio of SUA/Cr and a higher risk of all-cause mortality in female participants only (HR, 1.10; 95% CI, 1.02–1.18). The multivariable-adjusted HRs (95% CI) for all-cause mortality across quintiles of baseline SUA/Cr were 1.28 (0.91–1.80), 1.00, 1.38 (0.95–1.98), 1.37 (0.94–2.00), and 1.57 (1.03–2.40) for male participants, and 0.92 (0.64–1.33), 1.00, 1.04 (0.72–1.50), 1.56 (1.06–2.30), and 1.59 (1.06–2.38) for female participants. When the data were further stratified on the basis of age (< 65 or ≥ 65 years), body mass index (< 22.0 or ≥ 22.0 kg/m^2^), estimated glomerular filtration rate (< 60 or ≥ 60 mL/min/1.73 m^2^), and presence of SUA-lowering medication, trends similar to those of the full population were found in all groups.

**Conclusion:**

Baseline SUA/Cr is independently and significantly associated with future all-cause mortality among hypertensive patients.

**Supplementary Information:**

The online version contains supplementary material available at 10.1186/s40885-023-00235-8.

## Background

Hypertension is the leading cause of premature deaths worldwide and a significant contributor to global cardiovascular morbidity and mortality [1]. Undiscovered and untreated hypertension significantly increases the risk of developing cardiovascular, brain, and kidney diseases and accounts for about half of all deaths related to heart diseases and stroke [2]. This study examines the role of uric acid as a risk factor for all-cause mortality with a focus on hypertensive participants.

Uric acid, the final oxidation product of the purine metabolism in humans, is produced by the activity of xanthine oxidase. Research has shown that increased serum uric acid (SUA) contributes to mortality [3]. Yet, related results in the literature remain inconsistent, which can be attributed to differences in factors, such as sex, age, race, medication, underlying diseases, and stage of disease. Renal function appears to have a strong influence in these interactions [4]. While the level of endogenous SUA is primarily contingent on renal clearance function, and increased SUA often results from renal dysfunction [5], some previous studies have ignored the effect of renal function on SUA [6].

SUA normalized by renal function (SUA to creatinine ratio [SUA/Cr]), which has emerged as a new biomarker, is considered an excellent indicator of net SUA production and can be easily calculated without any addition using common biochemical markers. SUA/Cr has long been studied as a biomarker in populations with a high prevalence of chronic obstructive pulmonary disease [7], nonalcoholic fatty liver disease [8], and metabolic syndrome [9]. SUA/Cr has also been associated with cardiovascular events [10], and malignancy [11]. It is a predictor of incident chronic kidney disease and renal disease progression in type 2 diabetic patients with preserved kidney function [12, 13]. A recent study based on the National Health and Nutrition Examination Surveys showed that a higher SUA/Cr was associated with increased all-cause mortality among adults [11]. However, there is a dearth of research examining the relationship between SUA/Cr and all-cause mortality among hypertensive patients in the context of Asia, particularly Japan.

We therefore developed new indices using SUA/Cr to evaluate the relationship between baseline SUA/Cr and potential risk factors, such as hypertension, hyperglycemia, lipids, renal dysfunction, and all-cause mortality, using cohort data for hypertensive patients.

## Methods

### Study design and participants

This study is a prospective cohort analysis of data from the 2002 (cohort 1) and 2014 (cohort 2) Nomura Cohort Study [14]. Participants were rural residents of Seiyo city who had undergone community-based annual health examinations. A flowchart of participant enrollment and exclusion is presented in a previous study [14]. That study recorded the demographic and clinical metrics of age, sex, smoking habits, alcohol consumption, history of cardiovascular disease (CVD), and medical history, the data of which were collected using questionnaires. We conducted follow-up studies at 19-year intervals for the first cohort and at 7-year intervals for the second cohort. We obtained participants’ survival status from the Japanese Basic Resident Ledger. The present study focuses on individuals with hypertension, that is, individuals who reported systolic blood pressure (SBP) of 140 mmHg or higher, diastolic blood pressure (DBP) of 90 mmHg or higher, or were on antihypertensive medication. The first cohort comprised 1,606 participants and the second 665 participants. All participants were within the age range of 23 to 89 years. A total of 1,366 participants in the first cohort and 651 from the second underwent baseline physical examinations and participated in the follow-up study. We analyzed data for both cohorts (*n* = 2,017).

Follow-up surveys were administered at 19-year intervals for the first cohort and at 7-year intervals for the second cohort. Survival status was ascertained from the Japanese Basic Resident Register.

The study was approved by the Ethics Committee of Ehime University Graduate School of Medicine (No. 15,007,004). The study was also reviewed and approved by the Institutional Review Board of Ehime University Hospital (No. 1,903,018). All participants provided written informed consent.

### Evaluation of risk factors

We measured weight and height as baseline anthropometric indices. We calculated body mass index (BMI) as weight (kg) divided by height squared (m^2^). Smoking status (pack years) is the product of the number of years as a smoker and the average number of packs per day. Accordingly, we classified participants as nonsmokers, ex-smokers, light smokers (< 20 pack years), and heavy smokers (≥ 20 pack years). Similarly, we estimated daily alcohol intake on the basis of one bottle of sake (1 unit = 22.9 g ethanol). We categorized participants as nondrinkers, occasional drinkers (< 1 unit/day), light daily drinkers (1–2 units/day), and heavy daily drinkers (2–3 units/day). No participant consumed more than 3 units/day. We used an automated sphygmomanometer to measure blood pressure. Prior to the measurement, we asked participants to rest for at least 5 min. Participants remained in a seated position while an appropriately sized cuff was placed on their right upper arm to measure SBP and DBP. Participants also had to fast overnight before undergoing tests for their levels of triglyceride (TG), high-density lipoprotein cholesterol, low-density lipoprotein cholesterol, SUA, blood glucose (BG), and creatinine (Cr). The estimated glomerular filtration ratio (eGFR) was estimated by modifying the chronic kidney disease epidemiology collaboration equation with a Japanese coefficient as follows. Male: Cr ≤ 0.9 mg/dL, 141 × (Cr / 0.9)^–0.411^ × 0.993^age^ × 0.813; Cr > 0.9 mg/dL, 141 × (Cr / 0.9)^–1.209^ × 0.993^age^ × 0.813; female: Cr ≤ 0.7 mg/dL, 144 × (Cr / 0.7)^–0.329^ × 0.993^age^ × 0.813; Cr > 0.7 mg/dL, 144 × (Cr / 0.7)^–1.209^ × 0.993^age^ × 0.813 [15]. The following were classified as CVDs: ischemic heart disease, ischemic stroke, and peripheral vascular disease.

### Statistical analysis

We used IBM SPSS ver. 26.0 (IBM Corp., Armonk, NY, USA) to conduct statistical analyses. We denoted continuous variables showing a normal distribution as mean ± standard deviation and variables showing a non-normal distribution (e.g., TG and BG) as median and interquartile. We used log-transformed values for non-normally distributed parameters. Participants were divided into five groups according to the quintiles of baseline SUA/Cr. We performed a chi-square analysis to compare categorical variables and an analysis of variance on normally distributed variables to compare continuous variables. We performed multivariable analyses on the basis of the Cox proportional hazards using forced entry methods and age as the primary time variable. Furthermore, we compared the strength of the association between SUA/Cr, SUA, and Cr and risk of all-cause mortality estimated using the multivariable Cox proportional hazard model. Finally, we performed sensitivity analyses to determine if the observed association between baseline SAU/Cr and all-cause mortality was consistent. All *P*-values were two-tailed, and *P*-values below 0.05 were considered significant.

## Results

### Baseline characteristics of participants by quintiles of baseline SUA/Cr

Of the 2,017 participants, 916 (45.4%) were male. Mean age of the male participants was 67 ± 11 years and that of the female participants was 69 ± 9 years. Table 1 shows the baseline characteristics of the participants by quintiles of baseline SUA/Cr. The results indicated that higher BMI values, alcohol consumption, TG, SUA, SUA/Cr, and eGFR were significantly associated with higher quintiles of baseline SUA/Cr. Further, there was a significant association between lower age and Cr and higher quintiles of baseline SUA/Cr.

**Table 1:** Baseline characteristics by quintiles of baseline SUA/Cr (*n* = 2,017)

Characteristic	Quintile of baseline SUA/Cr					
	First (*n* = 406)	Second (*n* = 402)	Third (*n* = 403)	Fourth (*n* = 406)	Fifth (*n* = 400)	*P* for trend^a^
Male sex	202 (49.8)	193 (48.0)	166 (41.2)	194 (47.8)	161 (40.3)	0.015
Age (yr)	71.0 ± 9.0	69.0 ± 9.0	69.0 ± 9.0	66.0 ± 10.0	66.0 ± 10.0	< 0.001
Body mass index (kg/m^2^)	23.0 ± 3.0	23.6 ± 3.1	23.9 ± 3.2	24.3 ± 3.1	24.5 ± 3.5	< 0.001
Smoking status^b^						0.019
Nonsmoker	66.7	67.9	74.2	64.0	63.2	
Ex-smoker	15.1	16.4	11.2	14.8	16.8	
Light smoker (< 20 pack year)	8.2	4.7	6.0	5.7	7.2	
Heavy smoker (≥ 20 pack year)	10.1	10.9	8.7	15.5	12.8	
Drinking status^c^						< 0.001
Nondrinker	60.3	57.0	58.6	51.0	50.7	
Occasional drinker (< 1 unit/day),	23.5	24.6	19.1	21.7	20.5	
Daily light drinker (1–2 unit/day)	11.4	9.5	13.2	14.3	15.5	
Daily heavy drinker (2–3 unit/day).	5.0	9.0	9.2	13.11	13.3	
Cardiovascular disease	50 (12.3)	45 (11.2)	36 (8.9)	40 (9.9)	44 (11.0)	0.579
Systolic blood pressure (mmHg)	152.0 ± 16.0	150.0 ± 16.0	152.0 ± 17.0	149.0 ± 17.0	149.0 ± 18.0	0.120
Diastolic blood pressure (mmHg)	85.0 ± 10.0	85.0 ± 10.0	86.0 ± 10.0	86.0 ± 11.0	86.0 ± 11.0	0.257
Antihypertensive medication	215 (53.0)	233 (58.0)	216 (53.6)	225 (55.4)	222 (55.5)	0.646
Triglycerides (mg/dL)	97 (69–136)	90 (67–123)	97 (73–133)	99 (76–142)	107 (79–153)	< 0.001
High-density lipoprotein cholesterol (mg/dL)	62.0 ± 16.0	63.0 ± 15.0	63.0 ± 16.0	62.0 ± 16.0	63.0 ± 17.0	0.366
Low-density lipoprotein cholesterol (mg/dL)	116.0 ± 30.0	119.0 ± 29.0	122.0 ± 30.0	120.0 ± 30.0	120.0 ± 33.0	0.119
Antilipidemic medication	60 (14.8)	59 (14.7)	52 (12.9)	38 (9.4)	54 (13.5)	0.138
Blood glucose (mg/dL)	103 (93–117)	105 (93–120)	104 (94–118)	103 (93–117)	105 (95–119)	0.800
Antidiabetic medication	59 (14.5)	47 (11.7)	48 (11.9)	42 (10.3)	38 (9.5)	0.216
Creatinine (mg/dL)	0.86 ± 0.40	0.76 ± 0.18	0.70 ± 0.16	0.69 ± 0.15	0.62 ± 0.12	< 0.001
SUA (mg/dL)	4.20 ± 1.30	4.90 ± 1.20	5.20 ± 1.20	5.80 ± 1.20	6.30 ± 1.30	< 0.001
SUA-lowering medication	18 (4.4)	17 (4.2)	22 (5.5)	28 (6.9)	36 (9.0)	0.025
SUA/Cr ratio	5.00 ± 0.87	6.43 ± 0.30	7.40 ± 0.27	8.39 ± 0.33	10.20 ± 1.12	< 0.001
eGFR (mL/min/1.73 m^2^/yr)	65.00 ± 16.00	70.30 ± 12.50	74.30 ± 13.00	78.40 ± 13.70	84.60 ± 14.60	< 0.001

Data are presented as number (%), mean ± standard deviation, or median (interquartile range). The quintiles are distributed as the following: the first quintile, < 5.89; the second, 5.89–6.99; the third, 7.00–7.87; the fourth, 7.88–9.00; and the fifth, ≥ 9.01. Data for triglycerides and hemoglobin A1c were skewed and are thus presented as median (interquartile range) and were log-transformed for analysis

*SUA/Cr* Serum uric acid to creatinine, *SUA* Serum uric acid, *Cr* Creatinine, *eGFR* Estimated glomerular filtration rate

^a^Analysis of variance for continuous variables or the chi-square test for categorical variables

^b^Smoking status was defined as the number of cigarette packs per day multiplied by the number of years smoked (pack year)

^c^Alcohol consumption was measured using the Japanese liquor unit in which a unit corresponded to 22.9 g of ethanol

### Relationship between baseline SUA/Cr (continuous data) and characteristics

For the male participants, there was a significant positive correlation between SUA/Cr and alcohol consumption, DBP, TG, eGFR, and prevalence of SUA-lowering medication. However, there was a significant negative correlation between SUA/Cr and age, prevalence of antihypertensive and antidiabetic medication, and BG. For female participants, there was a significant positive correlation between SUA/Cr and BMI, smoking, alcohol consumption, prevalence of antihypertensive and SUA-lowering medication, TG, BG, and eGFR. However, there was a significant negative correlation with age (Table 2).

**Table 2:** Relationship between baseline SUA/Cr (continuous data) and characteristics by sex (*n* = 2,017)

Baseline characteristic	Male (*n* = 916)		Female (*n* = 1,101)		Total (*n* = 2,017)	
	*r*	*P*-value	*r*	*P*-value	*r*	*P*-value
Sex^a^	-		-		0.071	0.002
Age	–0.268	< 0.001	–0.146	< 0.001	–0.199	< 0.001
Body mass index	0.035	0.289	0.259	< 0.001	0.164	< 0.001
Smoking status^b^	0.058	0.078	0.167	< 0.001	0.031	0.158
Drinking status^c^	0.222	< 0.001	0.145	< 0.001	0.101	< 0.001
Cardiovascular disease	–0.017	0.610	0.003	0.908	–0.010	0.644
Systolic blood pressure	–0.033	0.314	–0.051	0.090	–0.039	0.076
Diastolic blood pressure	0.128	< 0.001	0.006	0.834	0.052	0.018
Antihypertensive medication^d^	–0.083	0.012	0.076	0.012	0.005	0.811
Triglycerides	0.097	0.003	0.106	< 0.001	0.099	< 0.001
High-density lipoprotein cholesterol	0.038	0.250	–0.014	0.654	0.020	0.380
Low-density lipoprotein cholesterol	–0.002	0.959	0.051	0.094	0.043	0.056
Antilipidemic medication^d^	–0.065	0.051	–0.006	0.832	–0.020	0.363
Blood glucose	–0.069	0.038	0.063	0.037	–0.008	0.721
Antidiabetic medication^d^	–0.081	0.015	0.034	0.256	–0.033	0.135
Estimated glomerular filtration rate	0.501	< 0.001	0.419	< 0.001	0.456	< 0.001
Serum uric acid lowering medication^d^	0.137	< 0.001	0.073	0.015	0.087	< 0.001

*SUA/Cr* Serum uric acid to creatinine, *r* Pearson correlation coefficient

^a^Male = 0, female = 1

^b^Smoking status was defined as the number of cigarette packs per day multiplied by the number of years smoked (pack year), and the participants were classified into nonsmokers (0), past smokers (1), light smokers (< 20 pack year, 2), and heavy smokers (≥ 20 pack year, 3)

^c^Alcohol consumption was measured using the Japanese liquor unit in which a unit corresponded to 22.9 g of ethanol, and the participants were classified into nondrinkers (0), occasional drinkers (< 1 unit/day, 1), daily light drinkers (1–2 unit/day, 2), and daily heavy drinkers (2–3 unit/day, 3)

^d^No = 0, yes = 1

### HRs and 95% CI of baseline SUA/Cr (continuous data) for all-cause mortality by sex

Table 3 lists the HRs and 95% CIs for baseline SUA/Cr for all-cause mortality in the univariable and multivariable analyses. For the female participants, baseline SUA/Cr was a significant predictor of all-cause mortality (HR, 1.10; 95% CI, 1.02–1.18). For the male participants, there was a significant inverse non-adjusted association between baseline SUA/Cr and the risk of all-cause mortality; however, this association was no longer significant after adjustment.

**Table 3:** Hazard ratios and 95% confidence intervals of baseline SUA/Cr (continuous data) for all-cause mortality by sex

Baseline characteristic	All-cause mortality	
	Hazard ratio (95% CI)	*P*-value
Male (*n* = 916)		
Nonadjusted	0.90 (0.84–0.95)	< 0.001
Age and eGFR-adjusted	1.05 (0.98–1.13)	0.148
Multivariate-adjusted^a^	1.05 (0.98–1.33)	0.157
Female (*n* = 1,001)		
Nonadjusted	0.98 (0.92–1.04)	0.511
Age and eGFR-adjusted	1.07 (1.00–1.14)	0.046
Multivariate-adjusted^a^	1.10 (1.02–1.18)	0.013
Total (*n* = 2,017)		
Nonadjusted	0.94 (0.90–0.98)	0.002
Age, sex, and eGFR-adjusted	1.07 (1.02–1.12)	0.008
Multivariate-adjusted^a^	1.08 (1.02–1.13)	0.004

*SUA/Cr* Serum uric acid to creatinine, *CI* Confidence interval, *eGFR* Estimated glomerular filtration rate

^a^Adjusted for baseline of all confounding factors in Table 2

### HRs and 95% CI for all-cause mortality in quintiles of baseline SUA/Cr by sex

**Fig. 1:**
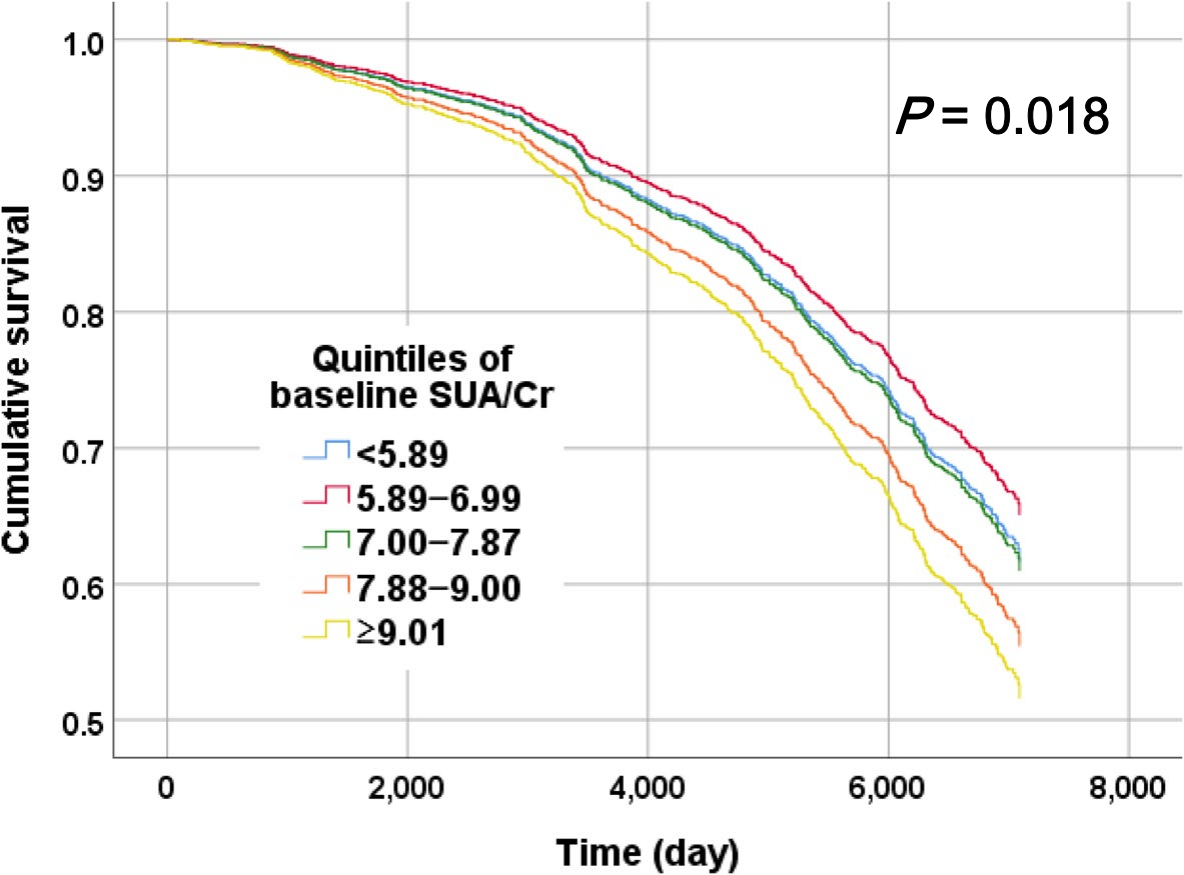
Cox proportional hazards model for all-cause mortality among hypertensive patients grouped by baseline serum uric acid to creatinine (SUA/Cr). Models were multivariable-adjusted for age, sex, and estimated glomerular filtration ratio

For both sexes, baseline SUA/Cr was a significant predictor of all-cause mortality. In particular, for the female participants, the fourth quintile (HR, 1.56; 95% CI, 1.06–2.30) and fifth quintile (HR, 1.59; 95% CI, 1.06–2.38) of SUA/Cr showed higher HRs than the second quintile (reference group). Figure 1 is a graphical representation of the cumulative mortality rates for the studied hypertensive patients after adjusting for age, sex, and renal function. The results indicated that the higher SUA/Cr group had significantly higher all-cause mortality than the lower SUA/Cr group (*P* = 0.018) (Table 4). The significant association with all-cause mortality disappeared when SUA and Cr were treated as the exposure variables (Table S1).

**Table 4:** Hazard ratios and 95% confidence intervals for all-cause mortality in quintiles of baseline SUA/Cr by sex

Baseline characteristic	Quintile of baseline SUA/Cr					*P* for trend
	First^a^ (*n* = 406)	Second^b^ (*n* = 402)	Third^c^ (*n* = 403)	Fourth^d^ (*n* = 406)	Fifth^e^ (*n* = 400)	
Male (*n* = 916)						0.020
Prevalence	89 (44.1)	57 (29.5)	65 (39.2)	64 (33.0)	52 (32.3)	
Nonadjusted	1.74 (1.24–2.42)	1.00	1.23 (0.86–1.75)	1.04 (0.73–1.48)	0.88 (0.61–1.28)	< 0.001
Age and eGFR-adjusted	1.35 (0.97–1.89)	1.00	1.33 (0.93–1.91)	1.43 (0.99–2.06)	1.60 (1.07–2.38)	0.186
Multivariate-adjusted^f^	1.28 (0.91–1.80)	1.00	1.38 (0.95–1.98)	1.37 (0.94–2.00)	1.57 (1.03–2.40)	0.277
Female (*n* = 1,001)						0.836
Prevalence	64 (31.7)	55 (26.3)	67 (28.3)	58 (27.4)	68 (28.5)	
Nonadjusted	1.08 (0.75–1.57)	1.00	0.90 (0.63–1.29)	0.91 (0.63–1.31)	1.04 (0.73–1.49)	0.786
Age and eGFR-adjusted	0.93 (0.65–1.33)	1.00	0.97 (0.68–1.39)	1.30 (0.89–1.89)	1.41 (0.98–2.05)	0.099
Multivariate-adjusted^f^	0.92 (0.64–1.33)	1.00	1.04 (0.72–1.50)	1.56 (1.06–2.30)	1.59 (1.06–2.38)	0.016
Total (*n* = 2,017)						0.030
Prevalence	153 (37.7)	112 (27.9)	132 (32.8)	122 (30.0)	120 (30.0)	
Nonadjusted	1.38 (1.08–1.76)	1.00	1.02 (0.80–1.32)	0.97 (0.75–1.25)	0.96 (0.74–1.24)	0.010
Age, sex, and eGFR-adjusted	1.13 (0.88–1.44)	1.00	1.15 (0.89–1.48)	1.37 (1.06–1.79)	1.54 (1.17–2.02)	0.018
Multivariate-adjusted^f^	1.09 (0.85–1.39)	1.00	1.19 (0.92–1.54)	1.42 (1.09–1.86)	1.61 (1.21–2.14)	0.010

Data are presented as number (%) or hazard ratio (95% confidence interval)

*SUA/Cr* Serum uric acid to creatinine, *eGFR* Estimated glomerular filtration rate

^a^202 Male and 204 female participants, < 5.89

^b^193 Male and 209 female participants, 5.89–6.99

^c^166 Male and 237 female participants, 7.00–7.87

^d^194 Male and 212 female participants, 7.88–9.00

^e^161 Male and 239 female participants, ≥ 9.01

^f^Adjusted for baseline of all confounding factors in Table 2

### HRs and 95% CI for all-cause mortality in quintiles of baseline SUA/Cr by subanalysis

Finally, participants were stratified by age (< 65 and ≥ 65 years), BMI (< 22.0 and ≥ 22.0 kg/m^2^), eGFR (< 60 and ≥ 60 mL/min/1.73 m^2^/yr), SUA-lowering medication (no and yes), and time to death (< 1,095 and ≥ 1,095 days) (Table 5). Overall, the results showed that the higher quintiles of baseline SUA/Cr (fourth and fifth quintiles) were significantly associated with a higher risk of all-cause mortality. For participants with eGFR less than 60 mL/min/1.73 m^2^/yr, we observed a significantly higher HR, forming a J-shaped curve, for the first and fifth quintiles compared with the second quintile. We obtained similar results for the analysis excluding those who died during the initial 3 years (< 1,095 days).

**Table 5:** Hazard ratios and 95% confidence intervals for all-cause mortality according to quintiles of baseline serum uric acid to creatinine (*n* = 2,017)

Baseline characteristic	No. of participants	Quintiles of baseline SUA/Cr Multivariable-adjusted hazard ratio (95% confidence interval)					*P* for trend
		First	Second	Third	Fourth	Fifth	
Age (yr)							
< 65	586	2.22 (0.89–5.56)	1.00	2.16 (0.88–5.34)	1.76 (0.72–4.26)	3.27 (1.35–7.88)	0.093
≥ 65	1,431	1.02 (0.79–1.32)	1.00	1.14 (0.87–1.49)	1.43 (1.08–1.90)	1.48 (1.09–2.01)	0.030
Body mass index (kg/m^2^)							
< 22.0	559	0.82 (0.55–1.23)	1.00	0.89 (0.56–1.41)	1.33 (0.82–2.14)	1.62 (0.99–2.67)	0.045
≥ 22.0	1,458	1.35 (0.98–1.86)	1.00	1.35 (0.98–1.85)	1.58 (1.13–2.19)	1.64 (1.15–2.34)	0.053
Chronic kidney disease							
eGFR < 60 mL/min/1.73 m^2^/yr	275	1.84 (1.10–3.08)	1.00	2.00 (1.10–3.61)	0.96 (0.42–2.20)	6.18 (2.27–16.8)	0.002
eGFR ≥ 60 mL/min/1.73 m^2^/yr	1,742	0.87 (0.64–1.17)	1.00	1.08 (0.81–1.44)	1.36 (1.02–1.82)	1.39 (1.02–1.88)	0.013
Serum uric acid lowering medication							
No	1,896	1.07 (0.83–1.38)	1.00	1.20 (0.92–1.57)	1.47 (1.11–1.95)	1.58 (1.17–2.14)	0.011
Yes	121	1.68 (0.48–5.92)	1.00	1.56 (0.47–5.21)	1.28 (0.44–3.78)	2.30 (0.69–7.70)	0.670
Time to death (day)							
< 1,095	50	-	-	-	-	-	-
≥ 1,095	1,967	1.11 (0.85–1.43)	1.00	1.18 (0.90–1.54)	1.42 (1.08–1.88)	1.57 (1.16–2.12)	0.025

Adjusted for baseline of all confounding factors in Table 2. The quintiles are distributed as the following: the first quintile, < 5.89; the second, 5.89–6.99; the third, 7.00–7.87; the fourth, 7.88–9.00; and the fifth, ≥ 9.01

*SUA/Cr* Serum uric acid to creatinine, *eGFR* Estimated glomerular filtration rate

## Discussion

This prospective follow-up cohort study aimed to examine all-cause mortality, as assessed by the Japanese Basic Resident Ledger, using data for 2,017 hypertensive persons, as well as potential confounding factors. The results indicated that baseline SUA/Cr, calculated using SUA and Cr, was significantly and independently associated with the all-cause mortality of hypertensive persons. In contrast, SUA or Cr alone did not predict all-cause mortality in hypertensive patients. To the best of our knowledge, few previous studies have indicated that baseline SUA/Cr as renal function-normalized SUA is an important risk factor for all-cause mortality among hypertensive persons [16].

SUA has a complex and paradoxical influence on the survival of hypertensive patients, and researchers have long debated its role in the context of mortality. SUA causes vascular endothelial damage [17] and induces hypertension [18, 19]. Numerous studies have also shown that SUA is associated with CVD outcomes and all-cause mortality in the general population [3, 20–22]. However, some studies have found a U-shaped association in this regard, suggesting that both low and high uric acid levels could increase mortality [20–23]. Interestingly, studies have presented similar results only for female participants [24, 25], male participants [26, 27], or mixed populations [23, 28]. While, A study reported that SUA was not an independent predictor of CVD or all-cause mortality in community-based type 2 patients [6]. This may be because SUA levels were primarily influenced by renal clearance function, wherein those with lower eGFR had higher SUA levels. In addition, renal dysfunction could be a major confounding factor in such studies [10].

SUA/Cr has been estimated using SUA and Cr, as well as SUA normalized by renal function, which has emerged as a new biomarker. SUA/Cr has been found to be superior to SUA or Cr in predicting all-cause mortality for elderly patients on hemodialysis [29]. Research examining the relationship between SUA/Cr and disease status and severity in patients with Parkinson’s disease has reported interferential effects of SUA on sex and renal function [30]. SUA/Cr has been associated with certain adverse health outcomes, such as metabolic syndrome in diabetic patients [31], postmenopause [9], β-cell function (i.e., insulin resistance) [32], renal dysfunction in diabetic individuals [12, 13, 16], and liver function in healthy subjects [33]. These outcomes are proven risk factors and may contribute to the pathology of all-cause mortality. The present study showed that a higher ratio of SUA/Cr predicted a higher risk of all-cause mortality among hypertensive patients. This association remained significant after adjusting for factors, such as sex, age, BMI, smoking and alcohol consumption status, history of CVD, blood pressure, BG, antihypertensive medication, lipids, antilipidemic medication, antidiabetic medication, eGFR, and SUA-lowering medication.

Researchers have yet to achieve a comprehensive understanding of mechanisms underpinning increased all-cause mortality in individuals with higher SUA/Cr. The biological mechanisms underlying this association are thought to include mainly oxidative stress, systemic inflammation (e.g., interleukin-1β, tumor necrosis factor-α, interleukin-6, and C-reactive protein) [34], and endothelial dysfunction [35] caused by prolonged high SUA levels. Recent studies have suggested that compared with postmenopausal women, premenopausal women have lower SUA levels. This is because estrogenic compounds increase the clearance of renal uric acid [36] and are strongly associated with other cardiovascular risk factors, such as age, sex, BMI, SBP, DBP, total cholesterol, TG, and BG [37]. Thus, the association between SUA and all-cause mortality largely reflects the predominance of metabolic risk factors, such as aging, insulin resistance, dyslipidemia, and renal dysfunction. These findings on the effect of SUA may not apply to male participants who exhibit such factors. The present study highlights a more prominent association between SUA and all-cause mortality in female participants than in male ones.

The main strength of this study is its accuracy, which can be attributed to the long-term study period including the follow-up analyses. Other advantages include a large sample size, adjustment for several possible confounding factors, and the inclusion of sensitivity analyses. However, the study was also subject to certain limitations. First, In the cohort analyzed in this study, the start dates of both are quite far apart, and it is necessary to take into account the effects of time bias, such as aging and associated differences in background diseases in the population. Second, the sample consisted of relatively healthy middle-aged and older adults (mean age, 68 ± 10 years) who participated in health examinations and lived in a rural area of Japan with a rapidly aging population. Thus, this sample may not be considered representative of the general population. Third, the high accuracy of the survey can be attributed to its focus on people whose deaths were registered in the Basic Resident Registry. These data excluded participants who moved out of the region during the survey period, constraining the potential for a subsequent follow-up study. Fourth, future research should consider the impact of changes in medications, underlying diseases, and lifestyle at the baseline and during the follow-up period. Fifth, we assessed renal function only on the basis of eGFR and not as per data on urinary protein or urinary albumin. Finally, the relatively small number of participants and deaths may have weakened the causal relationship between SUA/Cr values and all-cause mortality.

## Conclusion

This study demonstrated that SUA/Cr is strongly associated with all-cause mortality in hypertensive patients. Additional studies are needed to evaluate the reproducibility of our results and to elucidate the associations among the tested conditions further.

## Supplementary Information

**Additional file 1: Table S1.** Hazard ratios and 95% confidence intervals for all-cause mortality in quintiles of baseline SUA/Cr, SUA, and Cr (*n* = 2,017).

## Data Availability

The data that support the findings of this study were obtained from the Ethics Committee of Ehime University Hospital. However, restrictions apply to the availability of the data used under license for the current study, and they are, therefore, not publicly available. Data can be made available by the authors upon reasonable request and with permission from the Ethics Committee of Ehime University Hospital.

## Abbreviations

BG: Blood glucose
BMI: Body mass index
CI: Confidence interval
Cr: Creatinine
CVD: Cardiovascular disease
DBP: Diastolic blood pressure
eGFR: Estimated glomerular filtration ratio
HR: Hazard ratio
SBP: Systolic blood pressure
SUA: Serum uric acid
SUA/Cr: SUA to creatinine
TG: Triglyceride
