# Supplementary material for: Serum uric acid to creatinine ratio is a useful predictor of all-cause mortality among hypertensive patients
Source: Clin Hypertens. 2023 Apr 1;29:10. doi: 10.1186/s40885-023-00235-8 (PMC10067315; doi:10.1186/s40885-023-00235-8)
Supplement: Supplementary file 1 — Additional file 1: Table S1. Hazard ratios and 95% confidence intervals for all-cause mortality in quintiles of baseline SUA/Cr, SUA, and Cr (n = 2,017). [file 40885_2023_235_MOESM1_ESM.docx]

**Table S1.** Hazard ratios and 95% confidence intervals for all-cause mortality in quintiles of baseline SUA/Cr, SUA, and Cr (n = 2,017)

| Baseline characteristic | Quintile of baseline characteristics | | | | |  |
| --- | --- | --- | --- | --- | --- | --- |
|  | First | Second | Third | Fourth | Fifth | P for trend |
| SUA/Cr | 1.09 (0.85–1.39) | 1.00 | 1.19 (0.92–1.54) | 1.42 (1.09–1.86) | 1.61 (1.21–2.14) | 0.010 |
| SUA | 0.91 (0.70-1.17) | 1.00 | 1.15 (0.89-1.48) | 1.27 (0.98-1.66) | 1.15 (0.87-1.51) | 0.157 |
| Cr | 1.15 (0.52-2.51) | 1.00 | 0.86 (0.39-1.89) | 0.83 (0.38-1.83) | 0.72 (0.32-1.66) | 0.360 |

Adjusted for baseline of all confounding factors in Table 2. The quintiles of SUA are distributed as the following: the first quintile, <4.2; the second, 4.2–4.8; the third, 4.9–5.5; the fourth, 5.6–6.4; and the fifth, ≥6.5. The quintiles of Cr are distributed as the following: the first quintile, <0.61; the second, 0.61–0.65; the third, 0.66–0.70; the fourth, 0.71–0.84; and the fifth, ≥0.85.

SUA/Cr, serum uric acid to creatinine.
